# Effect of climate change on the potential distribution of *Helodermaalvarezi* (Squamata, Helodermatidae)

**DOI:** 10.3897/zookeys.1070.69186

**Published:** 2021-11-10

**Authors:** Aarón Gómez-Cruz, Nancy G. Santos-Hernández, José Alberto Cruz, Daniel Ariano-Sánchez, Christian Ruiz-Castillejos, Eduardo E. Espinoza-Medinilla, José A. De Fuentes-Vicente

**Affiliations:** 1 Laboratorio de Investigación y Diagnóstico Molecular (LIDiaM), Instituto de Ciencias Biológicas, Universidad de Ciencias y Artes de Chiapas, Tuxtla Gutiérrez, Chiapas, México Universidad de Ciencias y Artes de Chiapas Tuxtla Gutiérrez Mexico; 2 Red Mesoamericana y del Caribe para la Conservación de Anfibios y Reptiles, Tuxtla Gutierrez, Mexico Red Mesoamericana y del Caribe para la Conservación de Anfibios y Reptiles Tuxtla Gutiérrez Mexico; 3 Centro Universitario Tenancingo, Universidad Autónoma del Estado de México, México Universidad Autónoma del Estado de México Toluca Mexico; 4 Centro de Estudios Ambientales y Biodiversidad, Universidad Del Valle de Guatemala, Guatemala Universidad Del Valle de Guatemala Guatemala Guatemala

**Keywords:** Climatic change, beaded lizard, conservation, Chiapas, México

## Abstract

Climate change represents a real threat to biodiversity conservation worldwide. Although the effects on several species of conservation priority are known, comprehensive information about the impact of climate change on reptile populations is lacking. In the present study, we analyze outcomes on the potential distribution of the black beaded lizard (*Helodermaalvarezi* Bogert & Martin del Campo, 1956) under global warming scenarios. Its potential distribution, at present and in projections for the years 2050 and 2070, under both optimistic and pessimistic climate change forecasts, were computed using current data records and seven bioclimatic variables. General results predict a shift in the future potential distribution of *H.alvarezi* due to temperature increase. The optimistic scenario (4.5 W/m^2^) for 2070 suggests an enlargement in the species’ distribution as a response to the availability of new areas of suitable habitat. On the contrary, the worst-case scenario (7 W/m^2^) shows a distribution decrease by 65%. Moreover, the range distribution of *H.alvarezi* is directly related to the human footprint, which consequently could magnify negative outcomes for this species. Our research elucidates the importance of conservation strategies to prevent the extinction of the black beaded lizard, especially considering that this species is highly threatened by aversive hunting.

## Introduction

In the coming years, the impacts of climate change are projected to play a critical role in global biodiversity dynamics. Since 1980, rainfall and runoff levels at a global scale have decreased annually, which causes an overall prolongation of the dry season and affects entire biomes and ecosystems (Chou et al. 2012). The temperature of the planet has increased by approximately 1.1 °C since the pre-industrial period, and it is expected to have increased by 4.1 °C by the end of this century if current emission trends continue unchanged (Hidalgo et al. 2013; IPCC 2014; Masson-Delmote et al. 2018). In addition, deforestation, extractive activities, and the introduction of invasive species might contribute to the increasing negative effects on biodiversity (Durán et al. 2020).

Adaptative and geographic expansion responses of each group of organisms are crucial in determining their preservation or extinction. Accelerated global warming is causing the eradication of a significant proportion of the global reptile diversity (Sinervo et al. 2010; Durán et al. 2020). Due to their high species-richness, lizards constitute a major group of conservation concern (e.g., Sinervo et al. 2010; Diele-Viegas et al. 2020; Rozen-Rechels et al. 2020). In Mexico, research studies focusing on the effects of climate change on reptiles, and specifically on lizards, have been increasing in the last decade (e.g., Sinervo et al. 2017; Lara-Reséndiz et al. 2019, 2021; Domínguez-Guerrero et al. 2020; Gadsden et al. 2020). Nevertheless, studies relating to helodermatids are scarce.

*Helodermaalvarezi*, commonly known as the black beaded lizard, is one of the least studied Mexican lizards; hence, the impacts of climate change on this species are not well understood. However, the negative outcomes for helodermatids, given their sensitivity to temperature fluctuations, have been documented (Aranda-Coello et al. 2019; Ariano-Sánchez et al. 2020). For example, high temperatures may lead to fatal physiological alterations such as water loss and an elevated heart rate (DeNardo et al. 2004; Labra et al. 2008). In Mexico, the black beaded lizard has been reported in the Isthmus of Tehuantepec in the southern state of Oaxaca, the central lowlands of Chiapas, and on the border with Guatemala. Moreover, its presence was identified in the Nentón River Valley region of western Guatemala (Álvarez del Toro 1972; Ariano 2011). A lack of information makes it difficult to accurately determine the geographic distribution and conservation status of this species.

This study aimed to determine the potential geographical distribution of *H.alvarezi*, and to estimate the projected scenarios for 2050 and 2070 considering predicted climate change. Assessing the distribution of this species and the long-term environmental effects will allow us to understand its spatiotemporal variation, and to design effective conservation strategies for this species.

## Materials and methods

### *Helodermaalvarezi* point locality data

**Figure 1.**
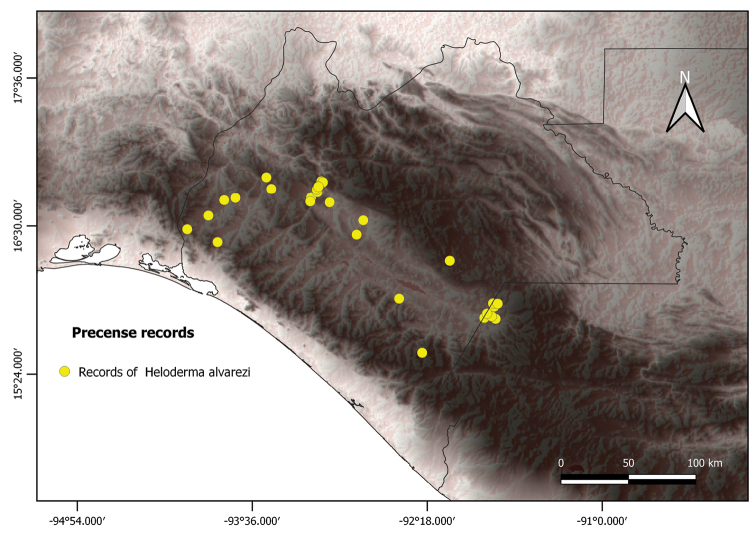
Presence records of *H.alvarezi* (yellow dots). Darker zones on grayscale indicate a higher altitude.

*Helodermaalvarezi* distribution data were collected between January and June 2020 from several bibliographic databases. These included GBIF (GBIF.org), Naturalista (naturalista.mx), VertNet (vertnet.org), and technical publications from the gray literature (Ariano 2011). To remove duplicate record entries and sampling errors, the data were filtered with the Wallace 1.0.6.1 software (Kass et al. 2018). Points were thinned to a distance of 1 km to avoid an overfitting in the modeling (e.g., Borzeé et al. 2019). In this way, a total of 27 records of *H.alvarezi* was obtained (Fig. 1).

### Climate modeling and prediction with all *H.alvarezi* distribution data

The Maximum Entropy (Maxent) approach (Philips et al. 2006, 2017) was used to model suitable potential areas for *H.alvarezi* under current and future climatic conditions based on presence-only observations of the species. According to the presence records of *H.alvarezi* used in our analyses, the biogeographical provinces Sierra Madre, Central Plateau, and Central Depression in Chiapas State, Mexico (Morrone 2019) were considered as the species’ accessible area (*M*) (Soberón & Peterson 2005). Climate modeling was performed at a 30 arc-second resolution with WorldClim2 climate data for 1950–2000 (Fick and Hijmans 2017). Pearson’s correlation coefficient was calculated for the 19 bioclimatic variables to evaluate multicollinearity, and to reduce uncertainty in the species distribution models (Elith et al. 2011; Cruz-Cárdenas et al. 2014). One variable was removed from any pair of variables with greater than 80% correlation, leaving seven bioclimatic variables to be used in the final models (Table 1). Ecological niche models were performed using the Maximum Entropy algorithm implemented in the Maxent v. 3.4.1 software. This software has been demonstrated to generate accurate predictions even when small sample sizes are used (Philips et al. 2006; Pearson et al. 2007; Martínez-Méndez et al. 2016).

**Table 1.** Bioclimatic variables and their contribution to model projections.

Acronym	Variable	Percentage contribution	Permutation importance
Bio10	Mean temperature of warmest quarter	31.1	34.2
Bio15	Precipitation seasonality	30	4.7
Bio17	Precipitation of driest quarter	20.9	46.2
Bio2	Mean diurnal range	15.4	12.6
Bio3	Isothermality	1.9	0.2
Bio4	Temperature seasonality	0.7	1.9
Bio18	Precipitation of warmest quarter	0.1	0.3

Niche models were projected to future scenarios for the years 2041–2060 and 2061–2080 using a GCM MIROC 6 model under different Representative Concentration Pathways (RCPs). Models were evaluated under simulated radiative forcings of 4.5 and 7 W/m^2^ to develop optimistic and pessimistic climate change forecasts, respectively (Lara-Reséndiz et al. 2019; Pérez 2020). To determine anthropogenic impact on the distribution of *H.alvarezi*, a carbon footprint estimation layer was overlaid on the niche models generated (Venter et al. 2016, 2018). The carbon footprint was obtained using cumulative emissions for the period 1993–2009, derived from population density, electrical power infrastructure, cultivable land areas, grasslands, roads, and railways (Venter et al. 2016, 2018).

Predictive accuracy was evaluated using the area under the curve (AUC). This can range in value from 0 to 1. A value less than 0.5 signifies that the classifier performs worse than a random classifier (Felicísimo et al. 2011; Mateo et al. 2011; Santillan 2013). The model with the highest statistical significance (AUC = 0.972) was selected. Model outputs were exported to QGIS 3.6.3 (QGIS.org 2020) and thresholded to 10% (Mejía et al. 2018), creating a binary distribution map where non-suitable areas are represented as zero (0) and suitable areas as one (1).

## Results

**Figure 2.**
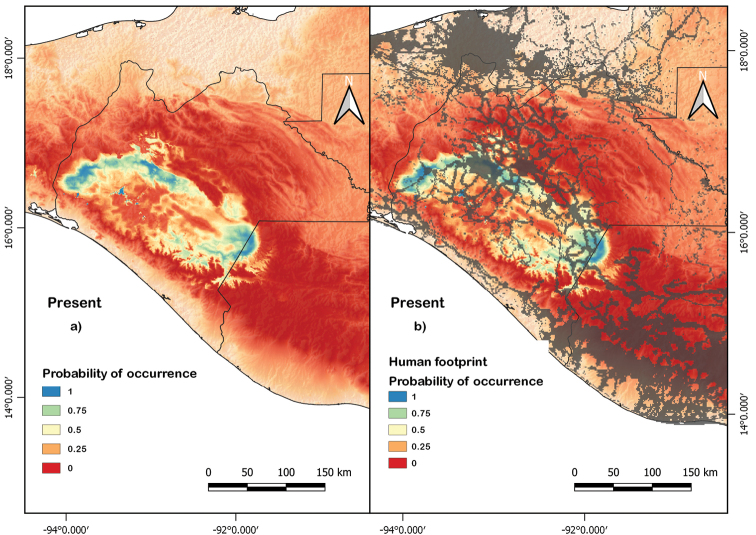
Current potential distribution model of *H.alvarezi* at present (**a**) and overlaid with human footprint (**b**).

The current potential distribution model covered a range of 11,218.63 km^2^, specifically across the Central Depression of Chiapas and the border with Guatemala (Fig. 2). The area of suitability increased at higher altitudes, from 400 to 1000 m a.s.l. Most of the distribution of the black beaded lizard is delimited by geographical barriers, such as the large mountain ranges that surround the valleys of the Central Depression in Mexico and Guatemala.

**Figure 3.**
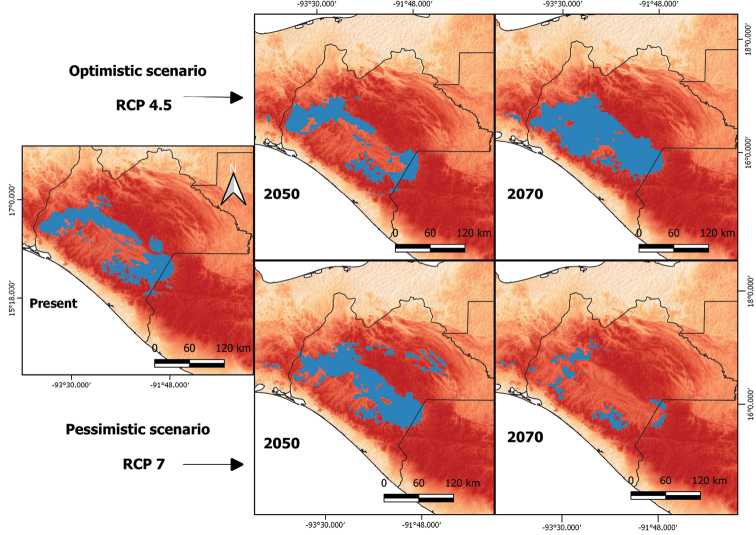
Potential distribution of *H.alvarezi* under different climate change scenarios using different Representative Concentration Pathways (RCP) and the seven bioclimatic variables that best explain the potential distribution of *H.alvarezi*.

Distribution models projected to the future for *H.alvarezi* show variations according to the case scenario and year. The distribution decreases by 12% for the optimistic scenario in 2050, compared with the current distribution. By 2070, the distribution is expected to increase by 61% with a displacement towards altitudes between 800 and 2000 m, mainly in the Sierra Madre de Chiapas. On the other hand, pessimistic scenarios for 2050 exhibit an increased distribution of *H.alvarezi* (32%), although predictions for 2070 reveal a reduction of 65.5% (Fig. 3).

## Discussion

According to our present-day distribution models, the distribution area of *H.alvarezi* extends over 11,218.63 km^2^ in the Central Chiapas Depression in Mexico and the Nentón River Valley in Guatemala. These areas are highly fragmented by human activity, which could induce individuals to settle where the impact of the human footprint is less pronounced, mainly on the borders of the Sierra Madre de Chiapas. This distribution excludes the regions of Socotelnango and La Concordia (Mexico) due to inappropriate environmental conditions (Suppl. material 1). Except for *H.charlesbogerti* (Campbell & Vannini, 1988), the distribution of *H.alvarezi* is the most restricted of any species in the Family Helodermatidae (Ariano 2007). The areas of high elevation in the Isthmus of Tehuantepec may act as a barrier that restricts the distribution of *H.alvrezi* in the direction of Oaxaca (Domínguez-Vega et al. 2012).

Our results suggest that *H.alvarezi* is found principally in seasonally tropical dry forests (SDTF), categorized as a highly threatened ecosystem (Trejo and Dirzo 2000; Trejo 2010). Over 30% of forest loss in Chiapas has been prompted by anthropic activities (Rocha et al. 2010). In addition to deforestation of the ecosystem, the aversive hunting of helodermatids may play an important role in their current distribution. Hunting intensity may be greater where the species distribution overlaps with the human footprint, although data are lacking. Historically, hunting, which threatens their distribution and abundance (Fig. 2a) (Aranda-Coello et al. 2019), has been carried out because of the appearance of these lizards and myths about their venom, as well as for the illegal skin trade.

Because projected distribution maps predict future implications of climate change on a species’ conservation status, these climatic forecasts enable us to take forward-thinking actions. The seven highest-contributing bioclimatic variables in the potential distribution modeling of *H.alvarezi*, in order, were: mean temperature of warmest quarter (Bio10), precipitation seasonality (Bio15), precipitation of driest quarter (Bio17) and mean diurnal range (Bio2). These are consistent with the findings of Domínguez-Vega et al. (2012) on the distribution of the genus *Heloderma*. It is important to mention that these models do not consider additional factors, such as aversive hunting (killing by local communities due to fear of their poisonous nature) (Domínguez-Vega et al. 2017). Hence, further research might examine these conditions.

When the human footprint layer was overlaid on the present-day distribution model of *H.alvarezi*, it may be seen that anthropogenic impact could be considerable. It has been observed with other organisms that human population growth, roads, and changes in land use affect an organism’s existence, especially by the occupation and transformation of the landscape, the alteration of the habitat and dispersal of physicochemical pollutants (Beck 2005; Colino-Rabanal and Lizana 2012; Pitts et al. 2017). However, not all species will have the same degree of exposure to the human impact, as this depends on taxon-specific ecological requirements (Colino-Rabanal and Lizana 2012).

According to our climate change scenarios, under an optimistic scenario (RCP 4.5) the distribution of the lizard would decrease by 2050, however, a substantial increase in projected distribution range is expected by 2070. This increase in distribution is expected towards the Sierra Madre de Chiapas, where perhaps suitable bioclimatic conditions for *H.alvarezi* are expected. Environmental factors (i.e., temperature and humidity) affect the species’ behavior and its habitat selection (Labra et al. 2008; Sinervo et al. 2018; Altamirano-Benavides et al. 2019).

As expected, the most unfavorable conditions to *H.alvarezi* will occur under a pessimistic scenario. This model projects a reduction in the distribution range by 65%; this percentage is higher compared to other *Heloderma*, such as *H.suspectum* (Giermanowsky et al. 2018), but similar to those of other lepidosaurs (see Güizado-Rodríguez et al. 2012). It is proposed that this scenario will lead to a decrease in precipitation and humidity, and would cause alterations in the species’ behavior. We know that the prolonged length of the aestivation period of these lizards ends with the arrival of the rainy period (Domínguez-Vega et al. 2012; Aranda-Coello et al. 2019; Ariano-Sánchez et al. 2020).

In addition, the rainy season begins the foraging and reproduction activity of the helodermatids, so a prolonged dry season would cause alterations in the population structure of these lizards (Ariano-Sánchez et al. 2020). Sinervo et al. (2010) suggested a probability of 91% for the extinction of Helodermatidae by 2080; this projection is consistent with our estimations for a pessimistic scenario. For some species, the process of adaptation to environmental changes is mostly slow and the ability to withstand climatic variations is limited (Foden et al. 2007). Rapid environmental changes and anthropogenic effects will be crucial to the survival of this species and could exacerbate the effects of range reduction. Analogously, the lack of knowledge about the thermal ecology of *H.alvarezi* in the wild is an important constraint on understanding its adaptability (Aranda-Coello et al. 2019). Therefore, it could be usefully explored in further research.

The implementation of conservation programs for species highly sensitive to climate change is fundamental. The present study suggests that the future of the black beaded lizard is not encouraging under different climate change scenarios. Promoting structural and functional connectivity among the remaining SDT patches and their associated mesic conditions is the most effective weapon to facilitate the altitudinal and latitudinal migration of the species under a global warming forecast. Additionally, slowing down climate change constitutes a primary focus on conservation status for the organisms living in xeric ecosystems, included *H.alvarezi*.

## Supplementary materials

Supplementary material 1 Supplementary figures and table Data type species data Explanation note **Figure S1.** Current potential distribution map of *H.alvarezi* with political boundaries. **Figure S2.** Future potential distribution of *H.alvarezi* with current human footprint projections. **Table S3.***Helodermaalvarezi* presence records used for potential distribution modeling. https://binary.pensoft.net/file/609119This dataset is made available under the Open Database License (http://opendatacommons.org/licenses/odbl/1.0/). The Open Database License (ODbL) is a license agreement intended to allow users to freely share, modify, and use this Dataset while maintaining this same freedom for others, provided that the original source and author(s) are credited.Aarón Gómez-Cruz, Nancy G. Santos-Hernández, José Alberto Cruz, Daniel Ariano-Sánchez, Christian Ruiz-Castillejos1, Eduardo E. Espinoza-Medinilla1, José A. De Fuentes-Vicente
